# Effects of non-invasive spinal cord stimulation on autonomic function in individuals with subacute spinal cord injury: A pilot clinical trial protocol

**DOI:** 10.1371/journal.pone.0347211

**Published:** 2026-04-20

**Authors:** Ryo Nakahara, Sarah Nasson, Ellie Bieler, Emilee Putsche-Young, Benjamin Aguila, Jiayi Shen, Emma Chung, Laura Winston, Stephen Burns, Deborah Crane, Margaret Eugenio, Klaus Krogh, Andrei Krassioukov, Chet Moritz, Soshi Samejima

**Affiliations:** 1 Department of Rehabilitation Medicine, University of Washington, Seattle, Washington, United States of America; 2 Amplifying Movement and Performance Laboratory, University of Washington, Seattle, Washington, United States of America; 3 Division of Gastroenterology, Harborview Medical Center, Seattle, Washington, United States of America; 4 Department of Hepatology and Gastroenterology, Aarhus University Hospital, Aarhus, Denmark; 5 International Collaboration on Repair Discoveries (ICORD), University of British Columbia, Vancouver, BC, Canada; 6 Division of Physical Medicine and Rehabilitation, Faculty of Medicine, University of British Columbia, Vancouver, BC, Canada; 7 GF Strong Rehabilitation Centre, Vancouver Coastal Health, Vancouver, BC, Canada; 8 Department of Electrical & Computer Engineering, University of Washington, Seattle, Washington, United States of America; 9 Department of Neurobiology & Biophysics, University of Washington, Seattle, Washington, United States of America; PLOS: Public Library of Science, UNITED KINGDOM OF GREAT BRITAIN AND NORTHERN IRELAND

## Abstract

**Introduction:**

Spinal cord injury (SCI) at or above the thoracic sixth spinal cord level disrupts descending sympathetic and parasympathetic control, leading to severe autonomic dysfunctions including cardiovascular and pelvic organ function. These complications adversely affect the quality of life and are associated with increased morbidity and mortality after SCI. Transcutaneous spinal cord stimulation (tSCS) may offer therapeutic benefits for these functions. The safety of tSCS in subacute SCI, however, remains unknown. Therefore, this study aims to evaluate the feasibility of tSCS for autonomic recovery in individuals with subacute SCI within six months since injury.

**Methods and analysis:**

We designed a two-phase clinical protocol consisting of a pilot randomized controlled trial conducted during inpatient rehabilitation (Project A), followed by a post-discharge outpatient phase with a single-arm quasi-experimental design (Project B). In Project A, 26 adults with cervical or upper thoracic (≥T6) American Spinal Injury Association Impairment Scale (AIS) A-C SCI are planned to be enrolled and randomly assigned to receive tSCS or sham stimulation for five sessions (up to 90 minutes each) in parallel with standard care. Following discharge from inpatient rehabilitation, eligible participants will be offered continuation in Project B. New eligible participants who have not participated in Project A will also be recruited into Project B. They will receive 18 tSCS sessions over six weeks in the laboratory setting. Primary outcomes focus on feasibility, including recruitment, retention, and stimulation-related adverse events. Clinical outcomes will be collected at baseline, after each intervention, and at six months and one-year post-injury. Feasibility results will be summarized descriptively, and exploratory analyses of autonomic outcomes, including cardiovascular and pelvic organ function, will provide preliminary estimates of autonomic responses.

**Ethics and dissemination:**

The study has been approved by the University of Washington Institutional Review Board. Written informed consent will be obtained from all participants. Results will be submitted to peer-reviewed journals and shared with the scientific/clinical communities and individuals with lived experience of SCI.

**Trial registration:**

ClinicalTrials.gov NCT06540859

## Background

Recent global estimates indicate that approximately one million new spinal cord injuries (SCI) occur each year, and more than 20 million individuals are living with SCI [1]. While paralysis is widely recognized as the most apparent and debilitating consequence of SCI, cardiovascular dysfunction is consistently reported as a leading health concern and the primary cause of mortality in this population [2–5]. Injuries at or above the level of sixth thoracic spinal cord segment (≥T6) disrupt descending supraspinal sympathetic control [6]. Loss of sympathetic outflow compromises vascular tone, which is essential for maintaining of arterial blood pressure [7].

Following SCI, the autonomic nervous system enters a state of neurogenic shock due to the loss of descending tonic sympathetic control and the dominance of parasympathetic activity [8]. Neurogenic shock typically lasts days to weeks following the injury onset [9]. During neurogenic shock, individuals with SCI experience profound hypotension and bradycardia, often requiring vasopressor support [10]. Although neurogenic shock itself eventually resolves, cardiovascular instability frequently persists beyond the acute phase because of loss of supraspinal regulation of autonomic spinal cord circuits below the lesion [11]. This impaired control manifests by variety of cardiovascular dysfunction including orthostatic hypotension, characterized by blood pressure (BP) reductions during postural changes [12], and autonomic dysreflexia, marked by excessive hypertension in response to visceral stimuli such as bladder and bowel distension [13]. Both orthostatic hypotension and autonomic dysreflexia can cause rapid and severe BP changes that are life-threatening [14].

SCI disrupts the crucial communication between spinal autonomic circuits and supraspinal control centers, resulting not only in impaired cardiovascular control but also in gastrointestinal and genitourinary system dysfunction [15]. These autonomic impairments are among the most important determinants of dignity, autonomy, and health [16,17]. Neurogenic bowel dysfunction results from impaired complex autonomic and somatic systems following SCI, and cardiovascular dysregulation during bowel management can induce clinically serious hypertensive episodes of autonomic dysreflexia, particularly in individuals with cervical and high thoracic SCI [18,19]. Therefore, there is a critical and urgent need for therapeutic approaches that extend beyond cardiovascular control and address multiple autonomic domains, including bowel and bladder function.

Engaging in targeted therapies during the acute and subacute phase, the critical window, may promote autonomic recovery [12,20]. Although sensorimotor function has been studied in the acute phase [21], only a few studies have focused on autonomic recovery. Evidence suggests that maintaining an optimal BP range during the acute phase promotes spinal cord recovery [22], and mean arterial BP management with vasopressor within one week of onset improves long-term neurological outcomes [23]. Pharmacological agents such as midodrine improve orthostatic tolerance in the acute phase [24,25]. Although midodrine may be effective for some individuals with SCI, its widespread use for treating orthostatic hypotension and orthostatic intolerance in this population remains questionable [26], and it may also exacerbate autonomic dysreflexia [27,28]. Prazosin can reduce autonomic dysreflexia severity but may induce a ‘first-dose phenomenon.’ Given the transient and fluctuating nature of BP in SCI, long-acting agents such as midodrine and Prazosin may not be ideal [12,29]. While topical nitropaste is also effective for acute management of autonomic dysreflexia, it has important limitations, including the risk of potential exacerbation of orthostatic hypotension and contraindications with phosphodiesterase-5 inhibitors [30]. In addition, non-pharmacological approaches such as verticalization in intensive care units have also shown long-term benefits [31]. However, the efficacy of interventions remains limited [32], and current clinical options are insufficient [12,29]. Therefore, there is an unmet need for novel therapeutic approaches that can prevent and target multiple dysfunctions while minimizing adverse effects.

Recent studies demonstrate that invasive epidural spinal cord stimulation (eSCS) can enhance autonomic function after cervical and upper thoracic SCI [33–36]. In acute and subacute SCI, animal studies in rodents and non-human primates demonstrate that eSCS can restore and maintain hemodynamic stability within hours after SCI [37]. Despite these benefits, the requirement for surgery and the associated costs restrict its broader application. In contrast, transcutaneous spinal cord stimulation (tSCS) offers a practical alternative that is non-invasive, less expensive, and allows electrode repositioning to target multiple spinal regions. Evidence suggests that tSCS engages spinal neural pathways in a manner similar to eSCS [38–40]. The real-time application of tSCS can potentially improve BP control and mitigate the episodes of autonomic dysreflexia in response to digital anorectal stimulation without delayed onset in individuals with chronic SCI [41,42]. Long-term application of tSCS has been shown to produce neurological and motor improvements that persist in the absence of stimulation in multicenter clinical trials [43].

In parallel, growing evidence suggests that recurrent tSCS may modulate autonomic cardiovascular regulation and neurogenic bowel dysfunction in chronic SCI with the absence of significant cardiovascular adverse events [44–46]. Clinical trials conducted in inpatient rehabilitation settings have demonstrated significant improvements in motor function, walking function, and functional independence at short-term follow-up [47]. Additionally, a recent study conducted in an inpatient rehabilitation setting examined the safety and feasibility of single-session, real-time tSCS for immediate blood pressure control in individuals with SCI [48]. However, there are no studies that have yet tested the effect of recurrent tSCS on comprehensive cardiovascular function including orthostatic hypotension and autonomic dysreflexia as well as pelvic organ function in subacute SCI, up to six-month post-injury. Thus, we aim to investigate the safety and feasibility of tSCS following acute SCI to treat a comprehensive range of autonomic dysfunctions.

Studying neuromodulation–based interventions in subacute SCI presents substantial ethical, logistical, and methodological challenges. Thus, transparent protocol-level reporting is therefore essential to ensure methodological rigor, mitigate selective reporting and publication bias, and enhance the interpretability and reproducibility of findings [49–51]. Publishing this protocol separately provides a pre-specified and transparent account of the study design, helping to mitigate selective outcome reporting and undisclosed analytical flexibility [52–54], while supporting scientific rigor and reproducibility through detailed documentation of stimulation parameters, titration procedures, and implementation strategies [55,56]. In accordance with the Standard Protocol Items: Recommendations for Interventional Trials (SPIRIT) guidelines, this protocol aims to provide a standardized and reproducible framework that may inform future trials incorporating rigorous randomized designs, appropriate sham controls, and standardized outcome measures, as well as clinical implementation of tSCS for autonomic recovery after SCI [57,58].

### Trial objectives

We hypothesize that tSCS can be safely administered during subacute SCI and will result in superior recovery of cardiovascular and pelvic organ functions. Our study is formed of the following aims: to assess the feasibility of single tSCS sessions in subacute SCI (Aim 1), to assess the feasibility of recurrent tSCS on autonomic function in subacute SCI (Aim 2), and to evaluate the preliminary effects of recurrent tSCS on autonomic recovery (Aim 3). These trial objectives and following study design are constructed based on the CONSORT statement and extension to pilot trials.

## Methods and analysis

### Study design and setting

**Fig 1:**
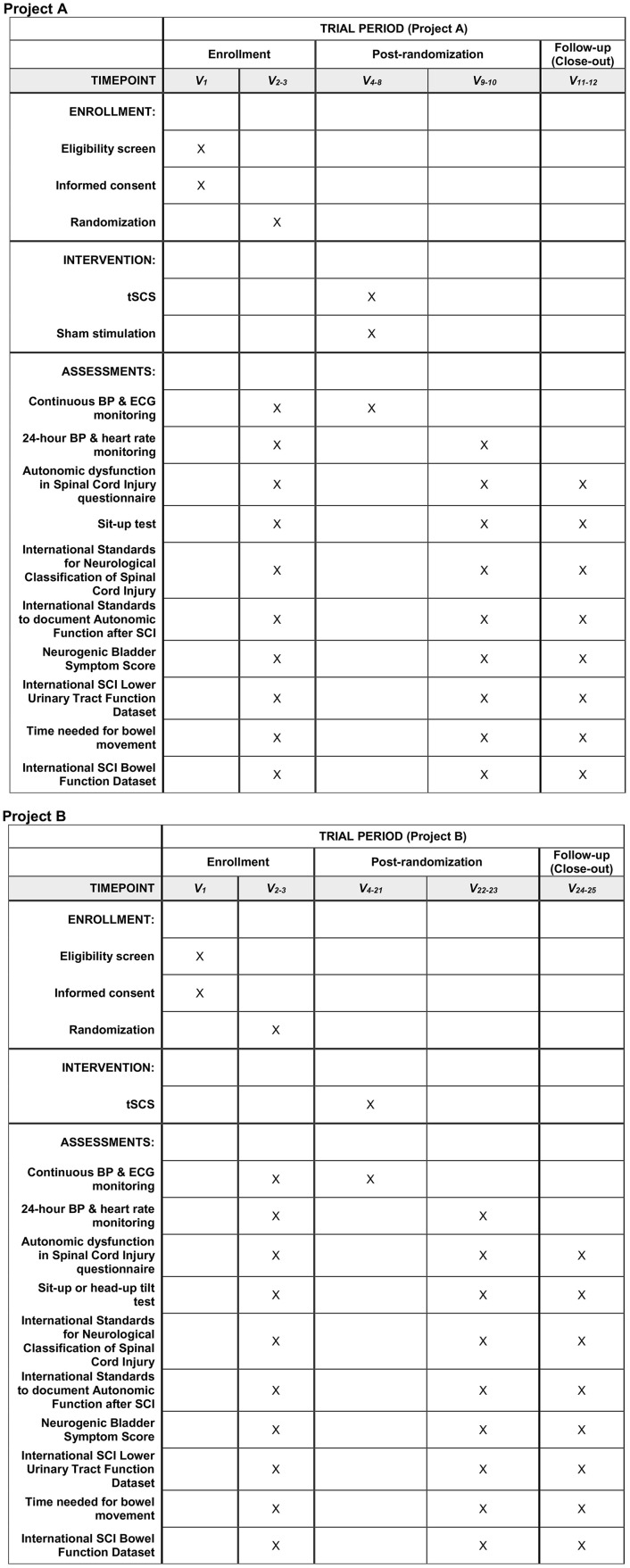
The standard protocol items: recommendations for interventional trials (SPIRIT) schedule: Enrollment, interventions, and assessments. V = Visit. tSCS = transcuntaneous spinal cord stimulation. ECG = Electrocardiography. BP = Blood pressure. SCI = Spinal cord injury.

**Fig 2:**
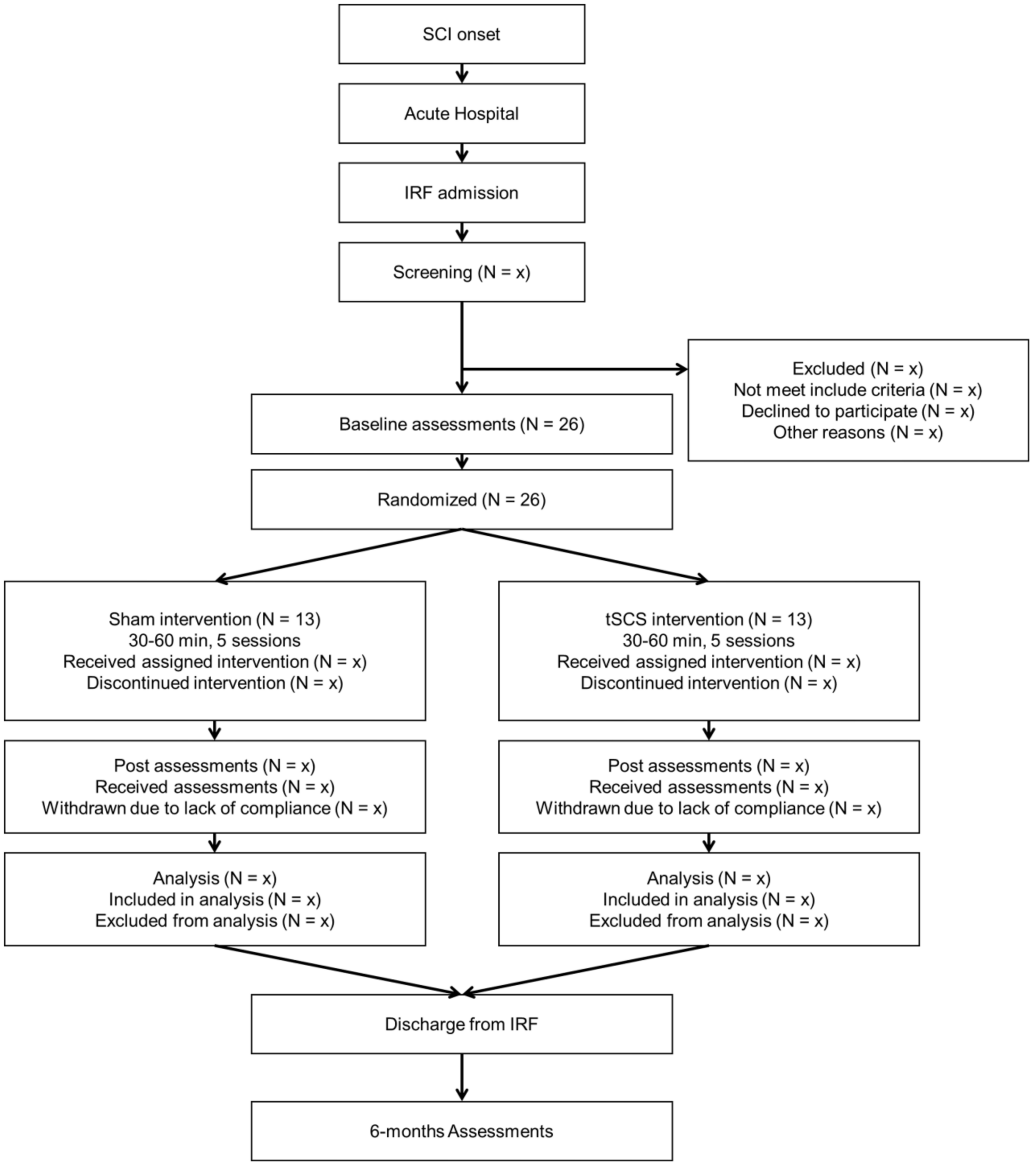
Trial flow and design of Project A. SCI = spinal cord injury. IRF = inpatient rehabilitation facilities. tSCS = transcuntaneous spinal cord stimulation.

The trial timeline, flow and experimental design is illustrated in Figs 1 and 2. We designed a two-phase clinical protocol consisting of a pilot randomized controlled trial (RCT) (Project A, Fig 2) followed by an outpatient continuation phase (Project B) designed as a within-subject, longitudinal feasibility study. Changes across a comprehensive range of autonomic dysfunctions are examined over repeated sessions, without a parallel control group, to reduce participant burden and allow inclusion of individuals transitioning from inpatient rehabilitation.

In Project A, we propose a pilot RCT with blinding subject and assessor, two-arm sham-control to evaluate the safety and efficacy of tSCS over the lower thoracic and upper lumbar spinal cord segments for cardiovascular function in adults (18–65 years old) with cervical and upper thoracic SCI (≥T6) American Spinal Injury Association Impairment Scale (AIS) A-C during subacute inpatient rehabilitation. We will recruit 26 individuals with SCI, admitted to inpatient rehabilitation facilities (IRF) (average 8–10 days post-injury). We will conduct this study in the University of Washington Harborview Medical Center (HMC).

Eligible participants will be randomly assigned into two groups as follows: One arm will receive sham stimulation for 30–60 minutes for a total of five sessions, during their IRF stay while maintaining intensive rehabilitation (Sham Group, n = 13). A second arm will receive tSCS for 30–60 minutes for a total of five sessions, in IRFs while maintaining intensive rehabilitation (tSCS Group, n = 13). The intervention frequency and duration are constrained due to the inpatient schedule. We will apply tSCS at rest while maintaining conventional physical therapy, occupational therapy and speech therapy to control for the combinatory effect. This study design also controls for the placebo effect by blinding participants, healthcare professionals, and assessors but not researchers who deliver the intervention. We will collect all outcomes over six months, with three assessments, one at each timepoint of IRF admission, post-interventions, and six-month follow-up.

Following discharge, participants who remain eligible and are able to commute will be offered enrolment in Project B. In addition, new eligible participants, who did not participate in Project A and are less than four months since the injury onset, will be screened and recruited into Project B. All participants who remain eligible from Project A, including those who received sham stimulation during Project A, will be eligible to participate. This project will utilize a single-arm quasi-experimental design in the laboratory setting. Participants will receive a total of 18 sessions of active tSCS for 60–90 minutes over six weeks to assess feasibility and longer-term autonomic adaptations. tSCS will be delivered at rest, separate from scheduled physical or occupational therapy sessions. All outcomes will be collected up to six months post-injury, with assessments conducted at three time points: baseline, after completion of the 18-session outpatient phase (Project B), and at six months follow-up. Participant recruitment for this study began on December 1, 2024, and is expected to be completed by September 30, 2026, with data collection for all study phases including six months follow-up anticipated to conclude by April 30, 2027. Final analyses and dissemination of the study results are expected by July 30, 2027. Written informed consent is obtained from all participants prior to enrolment. This study was approved by the University of Washington Institutional Review Board on May 6, 2024. This trial was registered at ClinicalTrials.gov (NCT06540859). The authors confirm that all ongoing and related trials for this intervention are registered.

### Sample size

We aim to recruit 26 participants with SCI for Project A admitted to IRF (average 8–10 days post-injury). Project B will include participants continuing from Project A as well as additional eligible participants recruited post-discharge. A sample size calculation was performed using G*power software [59] with power set at 0.80 and α at 0.05. We conducted the power analysis based on one SCS study for BP control assessed by head-up tilt test with and without eSCS (n = 4, cervical motor-complete AIS A or B) [33]. A minimum sample of 11 participants is required to detect a significant change in systolic BP during head-up tilt test (mean systolic BP change between stim-off and stim-on = 16.2 ± 12.6 mmHg, effect size = 1.29). Considering a potential 20% dropout rate [60], we aim to recruit 13 individuals with SCI to each group, for a total of 26 individuals with SCI.

### Recruitment

To control the effect of age, we will use 65-year-old cut-off considered as the conventional threshold for cardiovascular disease risk as indicated by the literature review of clinical trials. We aim to stratify participants based on relevant biological variables (e.g., age and sex) and secure a balanced representation of diverse demographic groups, thereby increasing the generalizability of our findings. Cardiovascular dysfunction becomes particularly prominent at neurological levels ≥T6. Such impaired control of the heart rate and blood vessels elevates ischemic risks and damages endothelial cells within the vessels, and fosters various vascular diseases, leading to increased mortality risk following SCI. Thus, our target population is 18–65-year-old adults with AIS A–C ≥ T6.

All subjects will be recruited from the inpatient rehabilitation program at the HMC. The screening will begin prior to admission to the inpatient rehabilitation program. The research assistant will follow the physicians’ referral for any potentially eligible patients. Those patients identified as potentially eligible will be approached in person by research study staff (coordinator or research assistant) in their hospital room during their first week of inpatient rehabilitation and request permission to both screen for and discuss the study based on approved screening form. People interested in volunteering will be evaluated for eligibility by one of the investigators and medical coordinators. Candidate participants will be asked for their oral consent to receive the screening questionnaires. This includes a yes-no questionnaire and multiple-choice questions in person. Inclusion and exclusion criteria are outlined in Table 1.

**Table 1:** Participant inclusion and exclusion criteria.

Inclusion criteria	Exclusion criteria
Are between 18–65 years of age. SCI (non-progressive) at or above the T6 spinal segment. Admitted to inpatient rehabilitation units or discharged from inpatient rehabilitation units but within 4 months since the onset of injury. American Spinal Injury Association Impairment Scale (AIS) A or B, or C for SCI. Have a stable medical condition that would permit participation in testing activities. Willing and able to comply with all clinic visits and study-related procedures. Able to understand and complete study-related questionnaires in English. Have no painful musculoskeletal dysfunction, unhealed fracture, pressure sore, or active infection that may interfere with testing. Are not currently pregnant or not intending to become pregnant during participation in this study. Are volunteering to be involved in this study. Must provide informed consent.	Autoimmune etiology of spinal cord dysfunction/injury. History of additional neurologic disease, such as stroke, MS, etc. Rheumatic diseases. (rheumatoid arthritis, systemic lupus erythematosus, etc.) Ventilator dependent. Clinically significant, unmanaged, depression, ongoing alcohol and/or drug abuse that interfere with study participation. Use of any medication or treatment that in the opinion of the investigators indicates that it is not in the best interest of the participant to participate in this study. Intrathecal baclofen pump. Cardiovascular, respiratory, bladder, or renal disease unrelated to SCI or presence of hydronephrosis or presence of obstructive renal stones. Presence of severe acute medical issues that in the investigator’s judgment would adversely affect the participant’s participation in the study. Examples include, but are not limited to acute urinary tract infections, debilitating pain, pressure sores, or unstable diabetes. Presence of pacemakers, stimulators, or medication pumps in the trunk, deep brain stimulators, metallic devices in the head such as aneurysm clips/coils and stents, or vagus nerve stimulators. A member of the investigational team or his/her immediate family. History of severe allergy. Malabsorption syndrome, primary hyperthyroidism, or hypogonadism. History of seizures.

### Transcutaneous Spinal Cord Stimulation (tSCS)

A portable tSCS device (SCONE and TESCoN, SpineX Inc., USA, ARC-EX, ONWARD Medical, Netherland) will be used to deliver biphasic 1ms pulses with 10kHz carrier frequency at 30 Hz. The device will be selected based on availability. This stimulation paradigm has demonstrated autonomic recovery in our previous studies [41,42,45]. The location of tSCS is predetermined at T11-T12 and L1-L2 vertebral levels. The stimulation intensity will range from 10 to 230 mA, and selecting the optimal therapeutic stimulation parameters will involve the following criteria: 1) a strong but comfortable sensory experience (initial slight tingling at the site of the electrode followed by some pressure or paraesthesia in skeletal muscles), described as a 5–7 on a 10 point Likert scale, 2) a stable systolic BP not more than 20 mmHg above baseline, and 3) without any symptoms of autonomic dysreflexia (i.e., sweating, goosebumps, tunnel vision, headache, etc.). The stimulation will be reduced if any of the above criteria is not satisfied as well if there are visually detectable skeletal muscle contractions. We will use two self-adhesive round electrodes (diameter of 2.0 cm) placed on the skin between spinous processes at the midline over the vertebral column as a cathode, and two rectangular 7.5  ×  12.5 cm self-adhesive electrodes located symmetrically on the skin over the iliac crests as anodes. The tSCS stimulator will be set with an open-loop stimulation using the same intensity throughout the session.

### Sham stimulation

Sham stimulation is designed to control for placebo effects associated with the perception of the intervention. Sham-controlled randomized designs are essential in neuromodulation research because improvements in autonomic function, pain and overall well-being are highly susceptible to placebo effects, participant expectancy, and observer bias [61]. Sham stimulation will be administered at the same anatomical location. Such sham stimulation has been successfully incorporated as the control treatment in previous and ongoing studies [62–64]. We have adapted these procedures to reduce the possibility that participants will be able to determine their group randomization. The intensity of electrical stimulation will be briefly ramped up to a level at which the participants report perceiving the stimulation (i.e., sensory threshold), then ramped down and turned off for the remainder of the intervention.

### Assessments

**Cardiovascular Monitoring.** Cardiovascular adverse events associated with tSCS at rest will be recorded. We will document the frequency and severity of abnormal BP responses, as well as cardiac responses including tachycardia, bradycardia, arrhythmia, and any associated symptoms. Twenty four-hour BP monitoring will also be used to track BP at day and night [65,66]. Participants will maintain a diary documenting daily activities such as sleep and wake, bowel and bladder management, taking medications, eating, and any instances of symptoms of autonomic dysreflexia and hypotension [67]. The monitoring involves wearing a compact digital BP device (Meditech Ltd., Budapest, Hungary), which is connected to a cuff around the upper arm. The device automatically measures BP at regular intervals, typically every 15 minutes during the day and 60 minutes at night. Heart rate variability will also be assessed. Time-domain and frequency-domain analyses will be performed to evaluate sympathetic and cardiac parasympathetic activity [68], in accordance with the European task force heart rate variability guidelines and based on our prior work [69]. Participants will fill out the Autonomic Dysfunction Following Spinal Cord Injury (ADFSCI) questionnaire at each assessment phase [70]. The ADFSCI questionnaire has participants self-report on the frequency and severity of autonomic dysreflexia episodes.

**Continuous Hemodynamic Monitoring During Orthostatic Challenge**. All participants will undergo continuous beat-by-beat BP monitoring and electrocardiography during a sit-up or head-up tilt test performed with and without tSCS to evaluate cardiovascular safety and real-time autonomic responses [71–73]. Orthostatic blood pressure responses are evaluated during transition from a supine to an upright position of at least 60 degrees [74].

**Neurological Function**. The International Standards for Neurological Classification of Spinal Cord Injury (ISNCSCI) [75] is used to determine the sensory and motor levels on both sides of the body, establish the overall neurological level of injury, and classify the completeness of the lesion (complete or incomplete). The ISNCSCI is a validated and standardized method for neurological assessment following SCI. The International Standards to document Autonomic Function after SCI (ISAFSCI) [76,77] provides a standardized framework for assessing key autonomic domains, including cardiovascular, thermoregulatory, sudomotor, bronchopulmonary, lower urinary tract, gastrointestinal tract, and sexual function, in conjunction with the ISNCSCI. The ISAFSCI will be administered at baseline and post-intervention to track changes in autonomic function along with the changes in neurological function.

**Lower Urinary Tract Function.** During each assessment phase, participants will complete a series of validated questionnaires to evaluate lower urinary tract function. These questionnaires include the Neurogenic Bladder Symptom Score [78], International SCI Lower Urinary Tract Function Dataset [79].

**Bowel Function.** Bowel function will be assessed using multiple validated measures. Time needed for bowel movement and the International SCI Bowel Function Dataset, which allows calculation of the Neurogenic Bowel Dysfunction score, [80] will be recorded at each assessment.

### Primary outcomes

The primary outcome for Aim 1 is the feasibility of tSCS in subacute SCI. Safety will be evaluated by cardiovascular responses before, during, and after single and repeated tSCS sessions, including changes in systolic and diastolic BP and heart rate, as well as the occurrence and severity of cardiovascular adverse events related to tSCS or study procedures.

The primary outcome for Aim 2 is the feasibility of recurrent tSCS during the inpatient rehabilitation phase (Project A), defined by (1) adherence to the stimulation and assessment protocols, (2) recruitment and retention rates throughout the study period, and (3) the frequency and severity of adverse events related to tSCS or study procedures. These feasibility indicators align with established guidelines for pilot and feasibility trials [81]. In addition, between-group differences in preliminary autonomic and cardiovascular outcomes will be explored following completion of the five-session inpatient phase (Project A). These outcomes include physiological and clinical measures of autonomic and cardiovascular function, such as systolic BP responses during the sit-up test, the frequency and severity of autonomic dysreflexia and orthostatic hypotension during 24-hour BP monitoring, and patient-reported symptoms of autonomic dysfunction assessed using the ADFSCI.

The primary outcome for Aim 3 is the change in systolic BP during orthostatic stress, assessed using the sit-up test or head-up tilt test, measured at baseline, following completion of the intervention, and at six-month follow-up.

### Statistical analysis

This is a two-phase clinical trial consisting of a pilot RCT (Project A) followed by a single arm pre- and post-experimental trial (Project B). A total of 26 participants with subacute (<six months since injury) cervical or upper thoracic ≥T6 AIS A–C SCI will be enrolled (Sham group n = 13, tSCS group n = 13). Descriptive statistics will be generated for the primary outcomes, feasibility outcomes, as well as demographic a clinical characteristic. Between group comparisons at baseline will be conducted using independent sample t-tests, Chi-square analyses, and Mann Whitney U tests as appropriate. Statistical analysis will be performed using R version 4.1.2 and the R studio program. This study is designed as a pilot trial, and analyses will primarily focus on feasibility, safety, and exploratory trends. Results will be interpreted in accordance with the CONSORT extension for pilot and feasibility trials

For Aim 1, we will evaluate the feasibility of tSCS by comparing cardiovascular responses before, during and after single and repeated tSCS sessions. Changes in systolic and diastolic BP and heart rate will be analysed using linear mixed models for repeated measures, with time (session) as a within-subject factor. Additionally, feasibility outcomes, including adherence rate, recruitment rate, and the frequency of adverse events, will be summarized descriptively.

For Aim 2, we will use Between-participant analyses assessing group differences in feasibility and preliminary autonomic outcomes between the Sham and tSCS groups after completion of the five-session inpatient phase (Project A). Linear mixed models will be used to assess the effects of time and intervention group, with baseline values included as covariates.

For Aim 3, within-participant analyses will examine longitudinal changes following the 18-session outpatient continuation phase (Project B) and at six-month follow-up. Descriptive statistics and standardized effect sizes (Cohen’s d) will be reported to estimate the magnitude of changes.

## Discussion and limitations

While spinal cord stimulation research is accelerating, few studies have assessed the applications of tSCS for use in inpatient settings and using a RCT design. Furthermore, there are no studies that have yet tested the effect of tSCS on comprehensive cardiovascular function including orthostatic hypotension and autonomic dysreflexia as well as pelvic organ function in IRF up to six-month post-injury. Thus, we propose to conduct the first study investigating the feasibility of tSCS following acute SCI.

### Data management and safety

All data collected (electronic or hardcopy documents) will be coded with unique identification numbers and stored centrally on the database, a password-protected computer, or in a locked filing cabinet in a secure laboratory space only accessible to the study investigators. To ensure information quality and accuracy after publication, all data will be stored for 6 years, and then subsequently destroyed.

Any adverse events reported during the intervention will be documented with information pertaining to their severity and anatomical location. The study coordinator will immediately report adverse events to the ethics board and Data and Safety Monitoring Committee (DSMC), which is comprised of three external, independent physician scientists with no involvement in the study, as well as the appropriate ethics board. The DSMC is responsible for safeguarding the interests of trial participants, assessing the safety and effect of the interventions during the trial, and monitoring the overall conduct of the clinical trial. The DSMC also provides recommendations for continuing or discontinuing the trial and outcome data use for participants who discontinue the trial. The trial progress will be biannually reported to the DSMC.

### Ethics and dissemination

This pilot study will be conducted in accordance with the Declaration of Helsinki and is consistent with the International Conference on Harmonisation Good Clinical Practice Guidelines, as well as applicable regulatory requirements. The study protocol was initially approved by the University of Washington Institutional Review Board (UW IRB) (STUDY00020243). The UW IRB and DSMC will be contacted when the trial makes any important protocol modifications. The feedback base on functional outcome measure results can be provided to participants for their adherence and benefits. The results of this pilot trial will be presented at national and international conferences and will be published in peer-reviewed journals. All subsequent manuscripts will be reported in conjunction with the Consolidated Standards of Reporting Trials. Extensive, individualized feedback will be provided to each participant upon completing the trial.

## Supporting information

S1 Checklist SPIRIT checklist is included as a supplemental document. (DOC)
